# One health’s “Jurassic Park moment”: tempered reasons for optimism

**DOI:** 10.1080/11287462.2025.2543098

**Published:** 2025-08-05

**Authors:** Jonathan Beever, Nicolae Morar

**Affiliations:** a Department of Philosophy and Center for Ethics, University of Central Florida, Orlando, FL, USA; b Department of Philosophy and Environmental Studies Program, Univerity of Oregon, Eugene, OR, USA

**Keywords:** One Health, Jurassic Park, interdependence, environmental bioethics, anthropocentrism, moral tension

## Abstract

In this short analysis, we argue that while One Health approaches have remained anthropocentric (i.e. morally and practically prioritizing human health), One Health is due for its “Jurassic Park moment.” Such a moment would mark a shift in moral priority, balancing human interests against nonhuman interests. Examples of theory and practice in One Health support the potential for such a shift.

In the space of bioethics and of public health (the intersections of which make up environmental bioethics, as we see it), our work is in support of a shift in imagination from people to planet. The value and goals of medicine can no longer be plotted only against successful procedures, fair access and the like, but need also to consider our planetary crisis. Both theorists and practitioners are growing aware of the fact we live in an interconnected or interdependent world, and thus the medical industry, from hospitals to pharmaceutical producers and practitioners, cannot overlook anymore their input into shared global concerns. One Health approaches, which engage interdependencies among human, non-human, and environmental health, have a powerful epistemic and ethical onus (Beever & Morar, 2018), yet their centrally anthropocentric implementations have left us with some pessimism.

Here, we argue that despite our pessimism the moral promissory note carried by the paradigm of One Health gives us reason for tempered optimism, as it may yet play an important role in our theoretical and practical work in environmental bioethics, understood as the ethics of relational conceptions of health across ecological scales.

Critics of One Health rightly point out the centrally anthropocentric yet important focus of its practical work. One could think here of the role of zoonotic diseases in global health in situations like the COVID-19 pandemic or closer to us in time, a potential avian flu. In spite of this current anthropocentric focus, we argue that One Health is facing its “Jurassic Park moment”: an ecologically-important shift in moral focus that will prioritize the value (and therefore welfare and health) of nonhuman individuals alongside human health.

Jurassic Park created an explosion of interest in paleontology, leading to a surge in academic and professional membership as that generation of viewers become students and practitioners – not only for discovery but also for the future of science. This “Jurassic Park moment” changed the momentum and direction of an entire discipline. There is a sense in which the One Health paradigm has become stuck as a service to human health, putting zoonotic and environmental science in the service of human health. Is One Health just waiting for its Jurassic Park moment? Such a moment could refocus and expand the moral priority to environmental or animal health – de-centering and reimagining human health as a truly relational function of these intersections.

Our optimism about One Health’s pivotal moment is driven by analogy with the generational transformation that we see in veterinary medicine, the founding discipline of One Health approaches. The moral ground of veterinary medicine has changed over time both practically and normatively, from a male-dominated anthropocentric emphasis on production animal health and zoonosis, to a female-dominated focus on companion animal health (see Buss, 2000) – or a change from farm practice to family practice. One Health’s history tracks this history of veterinary medicine from a focus on zoonosis in the service of human health to broader relational thinking about health and moral concern. The COVID-19 pandemic provided a conceptual and practical impulse for the roots of this change in thinking already embedded in those normative and demographic changes to the veterinary medicine profession. So we have hopeful reasons to think that, following changes in veterinary medicine as a leading discipline of One Health, the normative focus will also shift.

Scholarship on the science of One Health further bolsters our hope. Research in this space is offering evidence of the start of a normative shift by articulating turns toward solidarity with nonhuman others (Rock & Degeling, 2015), offering COVID-19-driven warnings of failures to connect the silos of One Health (Gruetzmacher et al., 2021), and analyzing the depth of interconnections among these silos (Cassidy, 2018). In synergy, a literature specifically on One Health ethics interdependent with the scientific literatures also supports this turn away from anthropocentrism. Calls for articulating One Health as a form of virtue ethics with an egalitarian ground (Capps, 2022), actively engaging value trade-offs (Lindenmayer et al., 2022), and reimagining moral claims in a world of interdependence (Beever & Morar, 2017) push against the massive momentum of anthropocentrism in One Health theory and practice.

Of course, our optimism must be tempered against our initial reasons for pessimism; for, other histories of One Health continue to prioritize complexity over breadth, marking out what seem to be exclusively human-focused problems like biosecurity, translational medicine, and scarce resources as the future “arena in which one health will be forged” (Bresalier et al., 2015, p. 13). Yet, from this history and scholarship we cannot help but see One Health as carrying a promissory note, conceptually and structurally, even if origins and current practices have largely ignored it. Cashing that note in can create new ways to think about the best path toward planetary health, and a centrally important moral expansion, especially in a turbulent anthropocentric world.

Does the promise of “One Health” suffice for our bioethical and public health practitioners to recognize genuinely and to respond appropriately to planetary tensions? The “One Health” framework certainly attempts to propose a unified view about “health,” where humans, non-human animals and the environment are interlinked and co-constitutive (Lederman & Capps, 2020). Albeit its intentions, it is worth considering whether this framework is *environmental* enough in order to generate fertile grounds for future environmental actions.

This is yet another place where our skepticism arises. One question certainly remains largely unanswered: what forms of valuation does One Health propose and, more importantly, how are we supposed to evaluate processes of value conflicts when significant forms of normative aggregation are at play? Certainly, if *human* interest *always* wins out, this framework could not sufficiently account for the value of the natural world without placing humanity *always* at the top of the moral hierarchy.

The case of nonhuman animal culling is the paradigmatic case in One Health analyses. In the face of the threat of zoonotic disease like avian flu, the go-to response is to destroy entire avian populations, in particular in factory farming settings. The reason for this is moral: the value of human individuals so greatly outweighs the value of avian individuals that even the mere threat of disease to the former is enough to warrant elimination of the latter. Despite this humanly-centered normative standpoint, calls for “zoonoethics” (Rodriguez, 2024) ask the hard questions about balancing out risks against harms. A small set of scholars like Chile’s Jayver Rodriguez are arguing for strengthened focus “against wildlife crime” and “crimes against biodiversity, not only as circumscribed and peripheral damages that affect human health but as damages that affect the health of humans, animals, and environments and constitute an attack against future generations” (Rodriguez, 2024, p. 21). Yet, we note, even Rodriguez is ambiguous as to how far that focus on crimes extends against threats to human health that still, for him, seem to take priority. In a UK context, Lederman and colleagues argue similarly that culling norms are the result of anthropocentric traditions more than scientific evidence (2021). Yet, like Rodriguez, they call for an “overall change in our normative attitudes toward animals and the environment” (Lederman et al., 2021, p. 26) without articulating how that moral balance shakes out.

Still, One Health holds this promissory note that gives us optimism. Perhaps it will yet have its own Jurassic Park moment.
